# In-field and abscopal response after short-course radiation therapy in patients with metastatic Merkel cell carcinoma progressing on PD-1 checkpoint blockade: a case series

**DOI:** 10.1186/s40425-018-0352-8

**Published:** 2018-05-30

**Authors:** Melody J. Xu, Susan Wu, Adil I. Daud, Siegrid S. Yu, Sue S. Yom

**Affiliations:** 1 Department of Radiation Oncology, Helen Diller Family Comprehensive Cancer Center, University of California San Francisco, 1600 Divisadero Street, Suite H-1031, San Francisco, CA 94115 USA; 2 Department of Medicine, University of California San Francisco, San Francisco, CA USA; 3 Department of Dermatology, University of California San Francisco, San Francisco, CA USA

**Keywords:** Metastatic Merkel cell carcinoma, Abscopal response, PD-1 inhibition, Single-fraction radiation therapy, Stereotactic body radiation therapy

## Abstract

**Background:**

Patients with metastatic Merkel cell carcinoma (mMCC) who experience disease progression on immunotherapy have limited additional standard options. Given evidence of synergism between radiation therapy (RT) and immunotherapy, two patients progressing on PD-1 inhibition were referred for short-course RT.

**Case presentation:**

Two patients were found to have progressive mMCC on PD-1 inhibitor therapy and were treated with single-fraction palliative RT. Both patients were observed to have local control at irradiated regions, as well as durable abscopal response at unirradiated, out-of-field, sites of metastatic disease.

**Conclusions:**

Short-course RT is a compelling strategy that could be a means to augment response in patients with mMCC who show progression on immune checkpoint blockade. Ongoing clinical trials are investigating the relationship between RT and immunotherapy in mMCC.

## Background

Merkel cell carcinoma (MCC) is a rare and aggressive skin cancer. Considered an immunogenic tumor with high expression of neoantigens, MCC has been identified as a promising candidate for immunotherapy trials [1–3]. Recently, two phase II trials for PD-1 and PD-L1 inhibition in metastatic MCC (mMCC) reported encouraging objective response rates of 56% in first line therapy and 32% in second line therapy [4, 5]. However, a small proportion of patients are either non-responders by 3 months or develop progressive disease. mMCC patients who have failed initial PD-1 immunotherapy have few additional treatment options, with chemotherapy conferring less than 8 months of progression-free survival (PFS) [6].

The abscopal effect, a phenomenon characterized by regression of untreated metastatic lesions following local therapy with radiation therapy (RT), is thought to be due to an immune stimulus mediated by improved antigen presentation and recruitment of CD8+ T cells after RT [7, 8]. A growing body of literature suggests an increased rate of the abscopal effect with the use of RT in combination with immune checkpoint inhibitors [9–12]. Previously felt to be a rare event following RT, the abscopal effect has been reported in up to 50% of patients with melanoma, even after progression on immunotherapy monotherapy [13]. While additional clinical trials are underway to investigate the effect of stereotactic body RT (SBRT) with immunotherapy in melanoma (NCT02821182, NCT02407171, NCT02659540) and non-small cell lung cancer (NCT02303990), the experience with mMCC is still developing.

We treated two patients who had progressive mMCC on immunotherapy and observed an augmented, out-of-field abscopal response after single-fraction palliative RT.

## Case presentation a

### Initial diagnosis and treatment

Mr. A was a 69-year-old man diagnosed with a T3N1bM0, stage IIIB right upper back MCC in 2014. He was treated with surgical excision and axillary lymph node (LN) dissection, with pathology demonstrating MCC. The tumor was positive for CK20 on immunohistochemical staining and Merkel cell polyomavirus was detected by polymerase chain reaction (PCR). The surgical margins were negative, but 2 of 29 LNs were found to be involved with cancer. Thus, he received adjuvant RT to a dose of 50 Gy in 25 fractions to the right axilla and posterior chest wall.

### Metastatic disease

A PET/CT two months later identified a hypermetabolic peripancreatic abdominal mass measuring up to 11.3 cm as well as a 1.1 cm left adrenal nodule. He was started on pembrolizumab. On CT of the abdomen and pelvis 10 weeks later, the mass had enlarged to 15.8 cm, encasing the celiac artery, hepatic artery, and splenic artery, with a new satellite omental nodule and two new enlarged para-aortic LNs. He began to develop symptoms of severe bloating and constipation. Due to the rapidly progressive and unresectable disease, he was referred for palliative RT.

### Radiation therapy

**Fig. 1:**
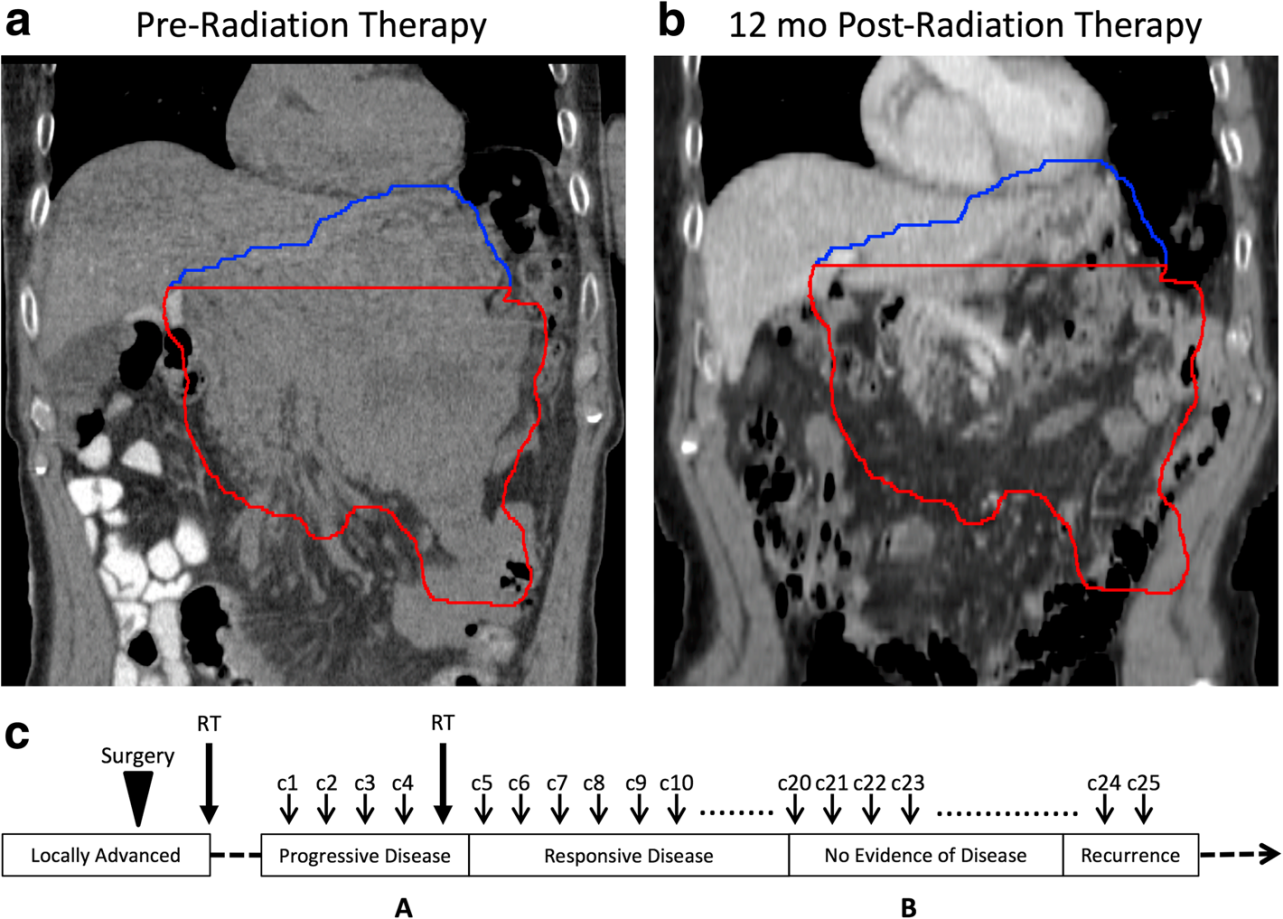
Case A. Abdominal metastasis before and after RT with target volume outlined in red and untreated disease outlined in blue. Left (Panel **a**): Coronal non-contrast CT scan obtained for RT planning demonstrating large abdominal mass. Right (Panel **b**): Coronal contrast CT obtained 12 months after RT demonstrating no evidence disease. Panel **c**: Timeline of therapy and disease status, with A and B corresponding to time points depicted in Panel **a** and **b** above. c1 = cycle 1 of pembrolizumab. CT = computed tomography. RT = radiation therapy

He received a single 8 Gy fraction of RT to the lower portion of the mass (Fig. 1 Panel A, red). The radiation field included the left adrenal nodule, omental nodule, and two enlarged para-aortic LNs. The superior portion of the tumor was untreated (Fig. 1 Panel B, blue). He tolerated the treatment well with near-immediate clinical relief and no acute toxicities. He continued to receive pembrolizumab.

### Treatment outcome

Two weeks after RT, a CT scan showed a decrease in his abdominal mass to 12 cm. At 2 months after RT, his abdominal mass had markedly decreased to 3.5 cm in size, with only residual pancreatic infiltration. By 8 months after RT, he only had minimal soft tissue involvement of the pancreatic body and tail. At 10 months after RT, he had no evidence of residual mass in the pancreas. At 12 months after RT, his PET/CT demonstrated no evidence of hypermetabolic malignancy (Fig. 1, Panel B). He was discontinued from pembrolizumab therapy and after stopping drug, was disease-free for another 17 months on surveillance scans. His most recent PET/CT detected a 2 cm isolated thyroid mass that was biopsied and confirmed to be mMCC. He has resumed pembrolizumab with RT to be given again based on response.

## Case presentation B

### Initial diagnosis and treatment

Mr. B was a 72-year-old man with a T1aN0M0, stage I left thigh MCC. He was diagnosed in 2015 by an excisional biopsy demonstrating MCC, which was CK20 positive. He had positive margins and subsequently underwent a wide local excision and sentinel LN biopsy, which showed no residual disease at the primary site and no LN involvement. He did not receive adjuvant RT.

### Metastatic disease

**Fig. 2:**
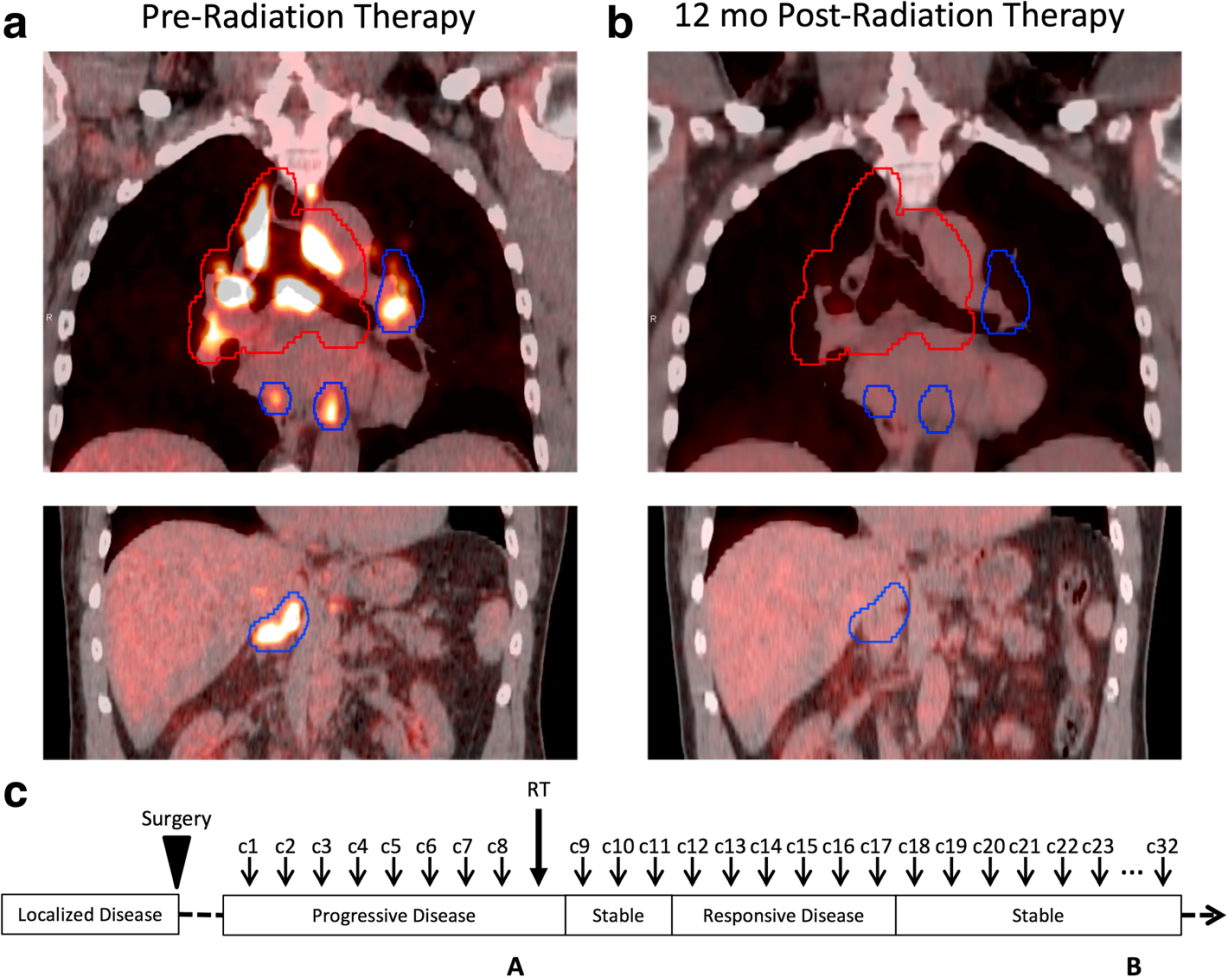
Case B. FDG-avid adenopathy before and after RT with target volume outlined in red and untreated disease outlined in blue. Left (Panel **a**): Coronal pre-RT PET/CT demonstrating mediastinal FDG-avid adenopathy in the mediastinum and bilateral hila (top) and upper abdomen (bottom); not pictured: supraclavicular adenopathy. Right (Panel **b**): Coronal PET/CT at 12 months after RT demonstrating radiographic response in the mediastinum and bilateral hila (top) and radiographic response in the upper abdomen (bottom). Panel **c**: Timeline of therapy and disease status, with A and B corresponding to time points depicted in Panel **a** and **b** above. c1 = cycle 1 of pembrolizumab. RT = radiation therapy. PET/CT = positron emission tomography/computed tomography. FDG = fludeoxyglucose

One year later, he noticed new left inguinal adenopathy, and needle biopsy revealed MCC. A PET/CT scan demonstrated bilateral hypermetabolic inguinal LNs and he was started on pembrolizumab. At a 3-month re-staging PET/CT, the level of fludeoxyglucose (FDG) uptake at his inguinal LNs improved but he had new extensive mediastinal adenopathy. He continued on pembrolizumab for an additional 2 months, after which a PET/CT demonstrated an increasing size and number (> 10) of hypermetabolic LNs in the supraclavicular, mediastinal, hilar, and upper abdominal nodal regions (Fig. 2). Due to the number of involved nodal regions, he was not a candidate for surgical resection and was referred for RT.

### Radiation therapy

He received a single fraction of 8 Gy of RT to his bulky mediastinal and right hilar lymphadenopathy (Fig. 2, top left). He tolerated the treatment well without acute toxicities and continued to receive pembrolizumab. His left hilar lymphadenopathy and abdominal and inguinal nodal disease remained unirradiated.

### Treatment outcome

At 2 months after RT, he had increased FDG avidity at his left inguinal LN but otherwise stable disease. At 4 months after RT, he had a complete response at his untreated supraclavicular and abdominal LN regions and decreased metabolism in the mediastinum, bilateral hila, and untreated left inguinal region. At 6, 9, and 12 months after RT, he had continued reduction and stabilization of hypermetabolism in his mediastinum, bilateral hila, and left inguinal region with no new areas of disease (Fig. 2, Panel B). He remains on pembrolizumab.

## Discussion

Immune checkpoint blockade is an exciting treatment option in mMCC, but a small proportion of patients do not respond or experience disease progression despite initial response. Herein, we describe two case examples whereby the addition of short-course RT was associated with subsequent improved immunotherapy response.

Both patients developed imaging evidence of disease progression with new and enlarging metastatic lesions within the first 3 months of initiating pembrolizumab. Pseudoprogression was considered in both cases. Pseudoprogression is a transient increased size of baseline tumor lesions due to edema and immune cell infiltration, and is occasionally used to describe delayed clinical responses in which initial increases in tumor burden are followed by tumor regression in the setting of immunotherapy [14, 15]. While pseudoprogression/delayed response has only been observed in two of 114 patients (1.8%) in the two phase II trials investigating pembrolizumab in mMCC, it has been reported to range from 2% in head and neck squamous cell carcinomas [16] to 6–7% in non-small cell lung cancer and melanoma [15, 17]. In patient A, pseudoprogression was unlikely due to the increasingly infiltrative nature of his growing metastasis, and a repeat scan in 3 months to rule out pseudoprogression was felt to be impractical given the high level of pain and need for prompt intervention. Patient B, who was found to have multiple new metastatic lesions increasing in size and number on serial imaging evaluations, was ruled out for pseudoprogression. Therefore, both patients were considered to have true disease progression.

In the setting of disease progression, standard treatment options are unfortunately limited and include cytotoxic chemotherapy or investigational trials. For the two cases described, single fraction RT was attempted for local control and to potentially enhance the opportunity for systemic response to continued pembrolizumab. These were the first two patients managed with this exact sequence at our institution and both achieved long-term local and systemic disease control.

We expected excellent local control and tolerability with single-fraction RT in mMCC. In a retrospective review of 26 mMCC patients treated with single-fraction RT of 8 Gy, 94% of patients had objective responses with a median PFS of 6.4 months [18]. Both patients tolerated their treatment course very well without any notable RT-associated toxicities. Consistent with prior reports of single-fraction RT, the treated sites of disease had marked response and durable local control.

Remarkably, both of our patients showed an out-of-field effect following immunotherapy and RT, with augmented response at unirradiated sites of disease. The timing of response, 2 months after RT for patient A and 4 months after RT for patient B, is consistent with what has been reported in the literature on abscopal effect, with a median time to response of 5 months [19]. Patient A’s untreated superior tumor demonstrated a complete response after 10 months, leading to pembrolizumab discontinuation at 12 months and a subsequent 17-month disease-free interval. In total, following RT patient A experienced a remarkable disease response for 29 months including a long treatment-free interval before developing a very recent 2 cm isolated thyroid recurrence. Patient B, who had diffuse LN involvement that was progressing prior to RT, now has a partial or complete response at multiple unirradiated LN regions at 12 months after RT. He remains on pembrolizumab with stable disease and continues to do very well clinically.

This is the first case series demonstrating an abscopal effect from limited-field, single-fraction RT in mMCC patients receiving immunotherapy. Two important clinical trials will investigate the interaction between immunotherapy and short-course RT in mMCC (NCT03071406 and Alliance A091605). Our case series supports the rationale of these trials. We believe there was synergism where the radiation may have acted as an in situ vaccine, enhancing the efficacy of the systemically administered immunotherapy [20, 21]. Interestingly, Patient A was an initial immunotherapy non-responder and Patient B was an initial responder with rapid re-progression, suggesting RT may have synergistic effects with immunotherapy in both patient populations.

We acknowledge that the findings of this case series require validation in larger, better-controlled cohorts and ongoing clinical trials. However, given that short-course RT is exceptionally well-tolerated, highly effective for local control, and potentially synergistic with immunotherapy, it is a compelling strategy that should be considered as a means to augment response in patients with mMCC who are progressing on immune checkpoint blockade. Although cure cannot be expected, our report demonstrates that a long disease-free and treatment-free interval is achievable with the addition of short-course RT in the setting of mMCC progression on immunotherapy.

## Conclusion

We report two cases of out-of-field abscopal response following single-fraction RT in patients with mMCC progressing on PD-1 checkpoint blockade. Ongoing clinical trials are investigating the synergistic effects of RT and immunotherapy in mMCC.

## Abbreviations

FDG: Fludeoxyglucose
LN: Lymph node
MCC: Merkel cell carcinoma
mMCC: Metastatic Merkel cell carcinoma
PCR: Polymerase chain reaction
PFS: Progression-free survival
RT: Radiotherapy
SBRT: Stereotactic body radiation therapy
